# Theory of Artificial Intelligence Adaptation for Linguistic Concordance: Bridging Gaps in Patient-Centered Communication

**DOI:** 10.7759/cureus.116343

**Published:** 2026-09-16

**Authors:** Razel B Milo

**Affiliations:** 1 Hahn School of Nursing and Health Science, University of San Diego, San Diego, USA

**Keywords:** ai healthcare translation, ai theory of communication, artificial intelligence, communication equity, communication in healthcare, language concordance

## Abstract

Background and aims

The use of artificial intelligence (AI) for healthcare translation remains underexplored, particularly in the literature, leading to a lack of evidence on how AI can help bridge language barriers between healthcare professionals (HCPs) and patients. This article introduces a new middle-range theory of healthcare communication designed to promote language concordance in AI-integrated healthcare environments.

Methods

Walker and Avant’s theory construction framework was used for theory synthesis, targeted literature review, and theory construction. Cumulative Index to Nursing and Allied Health Literature (CINAHL) and PubMed were searched using MeSH terms, including “artificial intelligence theory AND language concordance” and “artificial intelligence and language concordance.” The parameters used included full-text, peer-reviewed articles in English published within the past 10 years. A total of 163 abstracts were reviewed. Most articles were not AI-related and not related to language concordance. Eighteen relevant articles meeting the inclusion criteria were reviewed. The extracted key concepts were analyzed and synthesized into core constructs, followed by the construction of relational statements and theoretical propositions based on the logical connections among the synthesized concepts.

Results

AI Adaptation for Linguistic Concordance (ALiCon) is a middle-range theory that extends sociotechnical and communication theories. The key concepts, including language discordance, patient safety, HCP-patient relationship, patient engagement, improved clinical outcomes, and equitable technology (digital health and AI-driven technology), serve as the fundamental components of this theory, conceptualizing and operationalizing AI adaptation to achieve linguistic concordance in healthcare. Five propositions explain how these concepts interact with one another.

Conclusion

ALiCon theory advances nursing science by redefining technology and HCPs as mediators bridging communication gaps in healthcare. The rapid evolution of AI means the theory must be flexible as the technology advances.

## Introduction

The centuries-long increase in migration to the United States poses challenges for healthcare communication today. The United States Census Bureau reported that approximately 68 million individuals spoke a language other than English in 2019. This number has tripled since the 1980s, when it was 23.1 million. The most common languages spoken at home, aside from English, include Spanish or Spanish Creole, followed by Chinese, Tagalog (Filipino), Vietnamese, and Arabic [1]. Limited English Proficiency (LEP) means having difficulty speaking, writing, and reading English. Approximately 8.4% of United States residents are not fluent in English [2]. Given patients' linguistic diversity, healthcare professionals (HCPs) have a critical responsibility to address language barriers. A lack of language concordance (LC), or alignment between the patient's and HCP's language, can result in miscommunication, confusion, and diminished rapport. This leads to frustration for both parties. These challenges may also lead to mismanagement of disease and loss of follow-up, worsening health inequities in the United States. Previous literature has examined language discordance and how various technologies assist with interpretation between patients and physicians. However, few studies have integrated artificial intelligence (AI) technology into healthcare communication. Interest in AI in clinical settings has increased; existing literature focuses on technological accuracy or diagnostic performance rather than on the interpersonal dynamics of patient-provider interactions. Specifically, research on HCP-patient interactions remains limited.

Globalization, migration, aging populations, evolving societal dynamics [3], and increased language biodiversity shape global demographic change and threaten language loss in some areas of the world. Years of schooling is a stronger predictor of language loss than other socioeconomic factors. Many education systems do not support bilingual education, so children are not taught some languages. Predictive models indicate language loss in endangered areas is expected to triple over the next 40 years [4]. The United Nations Educational, Scientific and Cultural Organization (UNESCO) International Decade of Indigenous Languages is a global initiative to support diversity. Preserving language biodiversity is vital in healthcare practice. Technological advancements, including AI, give HCPs new opportunities to use their skills to support biodiversity in care communication. However, current AI algorithms in healthcare focus mostly on workflows, treatments, diagnostics, and data processing. The rapid adoption of AI in healthcare has outpaced our understanding of how to integrate communication dynamics into patient care, leaving HCPs without guidance. This article introduces a new middle-range theory of healthcare communication. It aims to promote LC in AI-integrated healthcare environments by establishing a framework that describes the operational, relational, and clinical systems that manage AI-mediated healthcare communications.

## Materials and methods

The theory of AI Adaptation for Linguistic Concordance (ALiCon) is a middle-range theory to guide healthcare research, practice, education, and policy. It integrates foundational ideas about linguistic concordance and AI adaptation. The theory addresses communication challenges arising from global demographic changes. Ethical considerations for AI-driven language translation mainly involve patient safety, translation accuracy, equity, regulatory requirements, and the HCP-patient relationship. This article discusses these ethical considerations further. The theory synthesis integrated concepts grounded in current evidence to inform knowledge about AI use and healthcare research, aiming to advance health equity by bridging health disparity gaps.

Middle range theory development

Theory development brings together different theories to generate greater insights. A systematic approach helps deepen understanding of relevant concepts in theories. This facilitates a comprehensive appraisal. One primary goal of theory synthesis is to develop a more systematic approach to generating theoretical thought. This approach consolidates concepts and unifies their relationships, making theory construction clearer. 

ALiCon was developed using Walker and Avant’s theory synthesis. An important aspect of this approach was integrating concepts grounded in theory and literature. This ensured the relevance and applicability of AI and LC in patient-centered communication [5]. The first step was to define the central concepts for theory synthesis, followed by a literature review. The literature review provided a clearer understanding of the relationships between the current research and the central concepts. This step strengthened the theoretical assumptions about AI and LC in healthcare. The last step was to organize the concepts and integrate them as a middle-range theory. 

Step 1: stipulating central concepts

The process began with a review of relevant theories, models, and frameworks. This provided a foundation for robust theory development. Several reviewed concepts were from nursing, social science, informatics, medicine, and AI. Some were dated and applied to different technology-related theories and patient-centered communication. The significant concepts were examined to find shared characteristics and potential relationships.

Sociotechnical theory (ST), developed by Trist in 1951, states that technical and social contexts must work together to improve quality and efficiency. In 1981, the framework was expanded to emphasize joint optimization, innovation, collaboration, commitment, internal controls, and a participative management style [6]. ST aligns with General Systems Theory (GST), introduced by Ludwig von Bertalanffy in the 1930s. GST integrates structural and functional components interacting with the environment. To maintain equilibrium, a system must regulate its inputs and outputs and adapt to environmental changes [7]. 

Technology-Enhanced Wisdom (TEW), a middle-range theory, informs the proposed framework. TEW seeks to advance healthcare education, research, and practice by supporting AI-driven information in healthcare. TEW explains how AI transforms the data-to-wisdom continuum, while emphasizing that human wisdom and judgment remain central to practice and ethics. AI tools augment healthcare technology and foster collaboration under human oversight; they do not replace human expertise. The mediating concepts within TEW shaped the key ideas in the proposed theory [8]. Crystallization Theory supports using AI to integrate multiple perspectives, including Positivist, Critical-Interpretive, and Pragmatic-Analytical approaches. In this model, humans handle high-level navigation and synthesis, while AI manages pattern recognition and evidence gathering [9]. The AI-Based Argument Theory in Patient Education looks at interactive dialogue systems to minimize information overload. For example, the Juvenile Idiopathic Arthritis Dialogue-based Education model turns patient education content into intuitive dialogues delivering high-quality information [10].

The Communication Accommodation Theory (CAT) explains how people communicate and engage with others [11]. This theory is used across cultures, languages, and disciplines. It guided practical interventions and training in healthcare. Giles and colleagues [12] described the theory’s growth from convergence and divergence to include intergroup identities, mediating mechanisms, and subjective outcomes that affect social results. They introduced stage 7, which addresses human-machine communication. It examines how people adapt and interact with technology or AI. CAT is crucial for how humans manage social distance and their relationships with automated environments. 

AI technology has greatly changed how we communicate. The theory of AI-mediated communication (AI-MC) uses technology to improve personal interactions [13]. A major challenge for this theory is that AI technology evolves quickly, often faster than effective communication or social strategies. This is also reflected in computer-mediated communication theory [14]. Technology must also align with cultural norms to be accepted, as described by the Technology Acceptance Model (TAM) [15]. When accepted, technology builds patient trust. AI in healthcare communication involves several key considerations. These include transparency about the technology, addressing ethical concerns, and potential AI algorithm biases. Data and human biases both influence AI, so a scientific approach is needed to build these algorithms [16]. This approach reduces bias and ensures scientifically sound results. AI technology is meant to enhance communication, not replace human connection. Human oversight is also crucial to maintain balance in AI integration and provide reassurance and confidence. Collectively, these main concepts extracted from the literature helped shape the proposed constructs. It meets the growing need for linguistically relevant AI technologies that advance and influence patient care.

Step 2: literature synthesis 

A targeted literature synthesis was conducted between 2025 and 2026 to explore and understand relationships between the central concepts and the current literature, described in the context of AI adaptation for healthcare linguistic concordance. The author searched the Cumulative Index to Nursing and Allied Health Literature (CINAHL) and PubMed using MeSH terms, including “artificial intelligence theory AND language concordance” and “artificial intelligence and language concordance”. The search included full-text, peer-reviewed articles in English published within the past 10 years. The author excluded articles without full text, non-peer-reviewed articles, white papers, editorials, conference abstracts, and studies not relevant to the central concepts. A total of 163 abstracts were reviewed. Most articles were not AI-related and not related to LC (n = 75). Many AI-related articles focused on diagnostics (n = 25), clinical decision support and workflows (n = 15), training and education (n = 4), prognostic and predictive modeling (n = 8), data extraction (n = 4), and assessment (n = 3). Some of the LC articles focused on psychometric evaluation (11). The author reviewed 18 relevant articles that met the inclusion criteria (LC [14] and AI-related [4]). The literature synthesis identified two themes (LC and AI technologies) and detailed their relationships with key constructs discussed in the results.

Language concordance

LC is crucial to patient care. The primary assumption behind LC is that a shared language promotes clear communication, enabling patients to better understand their care [17]. Most studies have discussed the physician-patient language gap. Research on LC among other HCPs (nurses, social workers, physical therapists, and others) remains limited. LC in communication between HCPs and patients must be a research and practice priority. This lack of research creates systemic barriers by limiting shared, proficient language between patients and HCPs, increasing the risk of harm, and decreasing trust. 

A study in Canada discussed the intersection of linguistic barriers and hospital-related adverse events. Their findings revealed that patients whose primary language was neither English nor French had the highest risk of harm (unadjusted rate of harm, 9.16%). Although the results did not establish the relationship between these two variables (linguistic barrier and harm), they suggest linguistic barriers often intensify current vulnerabilities and institutional risks. For example, non-English- or non-French-speaking patients experienced post-operative complications such as pneumonia and urinary tract infection [18]. The prominent clinical risks emphasize the need for standardized communication interventions to ensure patient safety across all healthcare disciplines.

LC strengthens the patient-provider relationship, fostering trust and rapport [19]. It can improve communication, increasing patient engagement, treatment adherence, and satisfaction [20]. Moreover, language is intricately tied to culture and beliefs. When healthcare providers are culturally sensitive and competent in understanding their patients' needs and preferences, clear communication becomes more effective. Clear communication reduces the risk of misinterpreting patients' symptoms, helping prevent misdiagnosis and procedural complications. Ultimately, LC can improve health outcomes and promote health equity [21]. Hence, the HCPs' roles in promoting LC are vital.

Effective patient communication is a critical determinant of clinical accuracy and patient trust. While ad hoc interpreters can be helpful at times, a professional interpreter (the gold standard) is preferred. However, many systems use ad hoc proxies for convenience and cost, which can lead to miscommunication (language discordance) and fail to meet regulatory requirements for meaningful access [22]. LC is a multimodal approach to both digital and verbal channels. Healthcare digital management (patient portals, secure messaging, remote patient monitoring, and telehealth) for chronic conditions, along with conventional communication methods (mail or telephone), is insufficient to meet patient needs [23]. 

AI technologies

AI-driven technology involves coordinating multiple AI models, agents, or tools to manage complex workflows. This process uses artificial neural networks, which consist of interconnected neurons that process and transform data. Machine learning (ML) also automatically optimizes prompts. It is essential to recognize the significant influence of the model's input and prior output on AI responses, as well as the complexity of human language and communication, given their diverse structures and conventions. Deep neural networks process intricate information, supporting language-development algorithms through deep learning and contextual understanding of spoken and written words. The key to an AI language model lies in natural language processing (NLP), which allows machines to understand and generate human language. They analyze text data to identify patterns and relationships between words, phrases, and concepts. AI algorithms rely on vast training datasets of text, primarily sourced from the internet, and they estimate the probability of word sequences [24]. 

AI can generate responses and engage in conversations through AI language models, automatic speech recognition (ASR), text-to-speech (TST), and machine translation (MT) [25]. To handle human inputs, these systems must recognize spoken language and convert it into text, and potentially back to spoken language. This is where GenAI speech recognition (GSR) and language models excel, ensuring accurate spoken-language recognition and input synthesis. Examples include Google Translate (Google LLC, Mountain View, CA) and various virtual assistants [26]. However, in healthcare, the context-specific nature of human language poses challenges, and evidence supporting the successful integration of these technologies in healthcare communication and LC remains limited. LC underpins shared decision-making, supports high-quality care, and improves patient satisfaction. By incorporating professional interpretation, advanced readability standards, and equitable AI implementation, health systems can bridge the gap between demographic realism and clinical practice.

AI in translation and documentation is an emerging technology. It is a scalable solution to the global shortage of multilingual HCPs. Yet implementing it requires meticulous safeguards to avoid systemic errors. Existing AI translation platforms show promise in performance and accuracy. For example, RadTranslate effectively delivered standardized multilingual audio radiology instructions, advancing workflow efficiency [27]. However, research on the effectiveness of AI translation platforms for HCP-patient communication remains limited [28]. This literature synthesis identifies several key concepts, including language discordance, patient safety, HCP-patient relationship, patient engagement, improved clinical outcomes, and equitable technology (digital health and AI-driven technology). These concepts provide a foundation for conceptualizing and operationalizing AI adaptation to achieve linguistic concordance in healthcare. Table 1 summarizes the literature synthesis.

**Table 1:** Concept analysis of literature review E = excluded; I = included

Authors	Language Concordance	Provider-Patient Relationship	Patient Engagement	Trust	Improved Clinical Outcomes	Digital Health	AI-Driven Technology
Barbieri et al., 2023 [23]	I	E	E	E	E	I	E
Cano-Ibáñez et al., 2021 [21]	I	E	E	E	I	E	E
Chandrashekar et al., 2021 [20]	I	E	E	E	I	E	E
Chonde et al., 2021 [27]	E	E	E	E	E	E	I
Crossley et al., 2020 [29]	I	I	E	E	E	I	E
Daggett et al., 2023 [19]	I	I	I	I	E	E	E
Haskard-Zolnierek et al., 2023 [30]	I	I	I	E	E	E	E
Jang et al., 2021 [31]	I	E	E	E	E	E	E
Lor and Martinez, 2020 [17]	I	I	E	E	I	E	E
Luera et al., 2024 [24]	E	E	E	E	E	E	I
Moneus and Sahari, 2024 [25]	E	E	E	E	E	E	I
Nelson and Johnson, 2024 [26]	E	E	E	E	E	E	I
Patel et al., 2022 [32]	I	E	I	E	E	E	E
Popper-Giveon, 2021 [33]	I	E	E	E	E	E	E
Reaume et al., 2020 [18]	I	E	E	E	E	I	E
Rechenberg et al., 2021 [34]	I	I	I	E	E	E	E
Rosenbaum, 2019 [22]	I	E	E	E	E	E	E
Xue et al., 2019 [35]	I	E	E	E	I	E	E

## Results

The last step in Walker and Avant's proposed theory development was organizing concepts into structures and propositions. ALiCon is a middle-range theory that extends TAM, CAT, AI-MC, and TEW. This section explores the ideas and statements related to AI adaptation in healthcare communication.

Construct one includes the antecedents and environmental factors that drive construct two. The main drivers of construct two are global demographic shifts, language discordance, and resource shortages. These drivers established the need to develop the core process with AI-human integrative adaptation and functional collaboration. This core process is construct two and includes AI-enhanced communication and HCPs' wisdom and oversight, which mediate the drivers, outcomes, and systemic effects (construct three). Construct three has short-term and long-term outcomes. Short-term outcomes include improved LC, enhanced HCP competencies, and improved patient rapport. Long-term outcomes include decreased patient harm, improved clinical safety standards, enhanced patient adherence, improved health system delivery, and better access to care for all. The constructs serve as the fundamental components of this theory, while the propositions describe the connections among them. The propositions also explain how these constructs can be used in healthcare.

Proposition one discusses AI and HCPs as mediators. AI-enhanced communication facilitates the development of new competencies by translating complex healthcare information into insightful dialogues. HCPs can collect patient information more precisely and respond effectively to complex needs.

Proposition two is that interactions between HCPs and AI must address validation, judgment, and empathy, which significantly affect the accuracy and effectiveness of AI-assisted communication. A team of experts should oversee the development, testing, evaluation, and implementation of these models. This team should include researchers, data scientists, healthcare experts, and cultural/linguistic experts. AI directs pattern identification and evidence curation; human judgment and critical thinking are still central to clinical practice and ethical reasoning. AI is a tool to strengthen HCPs' capabilities, not a substitute for human expertise.

Proposition three is the effectiveness of AI-MC across populations. AI-driven LC is most effective when it integrates cultural considerations, since language is entwined with culture and beliefs, thereby reducing the risk of misinterpreting symptoms and cultivating an HCP-patient relationship. Additionally, cultural norms and transparency promote patient trust and acceptance.

Proposition four is promoting clear communication between patients and HCPs, improving patient understanding (LC), leading to improved patient engagement, enhanced treatment adherence, and better-quality health outcomes. In contrast, language discordance increases the risk of misdiagnosis and clinical harm, thereby exacerbating health inequalities.

Proposition five encompasses AI-integrated healthcare environments, in which both patients and HCPs must adapt their communication strategies to build relationships with AI. AI-driven LC must synchronize its inputs and outputs and adapt to demographic shifts to maintain stability and safeguard patient safety. It is a scalable, reliable alternative for LEP patients. 

Theoretical description

The ALiCon middle-range theory was developed by synthesizing the main concepts and their dynamic relationships. The main drivers are the antecedents and environmental inputs (global demographic shifts, language discordance, and resource shortage). The core process is the AI-Human integrative adaptation, which underpins the theory. It is the shared interface between the AI technology (NLP, MT, GSR, and ASR) and healthcare expertise. In this interaction, the HCPs perform high-level practical navigation, while the AI technology handles language pattern differentiation and evidence curation. The AI technology must align with cultural norms and uphold transparency, which fosters end-user trust (patients and HCPs). 

Successful implementation of ALiCon leads to near-term advances in clinical interactions, trust, and decision-making support, as well as improved HCPs' ability to use AI-driven technology and provide culturally and linguistically appropriate care. The long-term impact of successfully implementing this theory is clinical safety, increased patient engagement (adherence), health system efficiency, and meaningful access to care for diverse populations. AI-driven LC helps mitigate systemic inequities. 

In summary, environmental factors, language discordance, and resource scarcity drive demand for AI-human collaboration. They have a mutually beneficial relationship in which AI enhances skills, while expertise provides ethical oversight and alleviates bias. This collaboration produces LC, which mediates improved health outcomes and system efficiency. Figure 1 presents the theoretical model. 

**Figure 1:**
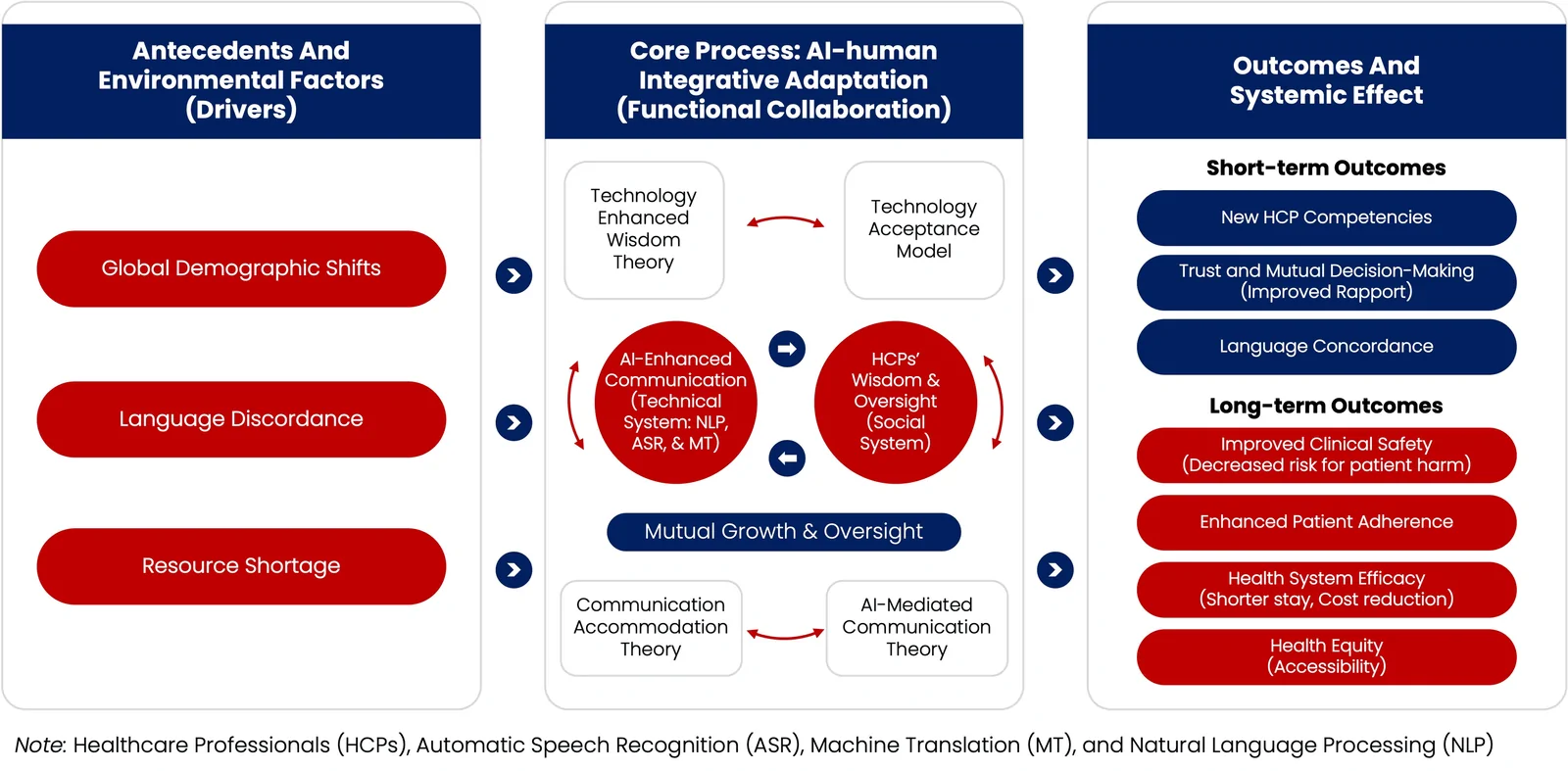
Theoretical model of AI adaptation for linguistic concordance Image credit: The author. Figure created using Microsoft PowerPoint (Microsoft® Corp., Redmond, WA).

Usability and testability

The ALiCon usability lies in clinical applications. This theory is designed for an AI-integrated healthcare landscape to ease the collection of pertinent patient information and efficient language translation. It transforms complex healthcare data into intuitive dialogues, enabling HCPs and patients to communicate effectively while delivering high-quality information. ALiCon supports theory- and evidence-based AI-driven translation and documentation tools. To ensure the technology's usefulness, ALiCon emphasizes AI-driven cultural alignment in translation, nurturing trust and enriching the HCP-patient relationship. 

ALiCon is a useful theory with practical applications for bridging communication gaps in healthcare. The empirical indicators are linguistic and process metrics. The LC metric can be measured by evaluating interventions and patient engagement. The AI operation leverages AI translation platforms (i.e., MT, ASR, and NLP) and analyzes intricate AI processes to identify language patterns and their relationships with words and concepts. It should be tested for clinical validity and reliability through psychometric evaluation. Bias can be measured by tracking declines in human and data biases in AI-generated replies. 

Primary metrics for success include reducing patient harm, improving the clinical accuracy of collected patient data, and preventing misdiagnosis through precise communication. Improvements in communication gaps are also measured by health system efficiency, such as reductions in hospital stays and costs. In addition, higher rates of home discharge rather than skilled healthcare facilities are a clinical indicator of efficient care coordination and patient recovery. 

The patient-centered metrics include patient satisfaction and trust, treatment adherence, shared decision-making, and technology accessibility. One subjective outcome is measured through evaluations of the HCP-patient relationship and acceptance of AI technology. Accepting and understanding AI technology enhances HCPs' technical competency and empowers them to use it in practice. Patients’ treatment adherence is the degree to which they follow the plan of care, a key indicator of effective communication and thorough shared decision-making with the HCP. The technology's accessibility is measured by the number of patients and HCPs using the AI-translation technology, as well as through continuous evaluation and improvement of its usability, efficiency, and effectiveness.

## Discussion

ALiCon theory advances healthcare science by redefining technology and HCPs as mediators bridging communication gaps in healthcare. As linguistic diversity rapidly increases, our conventional dependence on healthcare interpreters is no longer adequate to ensure patient safety and health equity. ALiCon is a structured, middle-range theory for navigating this change by incorporating sociotechnical concepts with the complexity of care. 

A primary contribution of ALiCon theory is highlighting AI's lack of capacity for ethical reasoning and empathetic validation. In the AI-human integration process, HCPs must remain the arbiters of clinical judgment. HCPs are essential to developing the AI-translation system, primarily because of their expertise and practical communication skills, including active listening, non-verbal communication, and patient-centered interaction [12]. Patient-centered care emphasizes therapeutic communication and cultural sensitivity and employs strategies such as active listening [11]. Effective implementation of AI technologies in healthcare communication largely relies on collaboration among HCPs, cultural and linguistic experts, and AI experts [10]. Unlike traditional communication models or technology models in which AI-assisted technology is a passive tool, ALiCon conceptualizes AI as an active, adaptive, dynamic mediator in clinical interactions between HCPs and patients. Its short-term and long-term outcomes will improve the healthcare communication landscape, reduce resource shortages, and decrease language discordance in healthcare. ALiCon specifically addresses how real-time AI translation alters relational dynamics, trust-building, and nuance retention in acute care dialogues, filling a critical gap left by general communication frameworks.

As we explore AI’s potential to handle complex datasets, it is vital to recognize HCPs' influence on the data used in the algorithm. To address AI biases, it is crucial to foster a culture of open discussion about the developers’ and HCPs’ predispositions. Their role is crucial in addressing potential barriers. This transparency and collaboration are key to ensuring the data used for AI models is free of bias, as this is vital to the technology's reliability and to building confidence in its application. For instance, a lack of cultural competence could lead AI models to overlook patients’ needs. Therefore, it is essential to consider and apply a sociotechnical context when working on these AI models and algorithms [6]. Additionally, HCPs can provide accurate information to inform AI algorithms and reduce bias by maintaining a rigorous approach to data collection, model and algorithm development, and by decoding and testing the AI system before launch [28]. By addressing these barriers, AI-powered language translation can reduce biases and help reduce systemic inequities experienced by LEP communities [20].

It is essential to address the technical and legal issues, including data privacy, data ownership, data access, and patient access to their medical records. These factors must be managed transparently to maintain users’ confidence, including both patients and HCPs, in AI-driven translation platforms [13]. During the planning phase, the team must clearly outline a comprehensive policy on AI data and privacy, ensuring patient data is protected from unauthorized access. Users must be educated on how to safeguard their data and maintain confidentiality [23]. Organizations should implement a multi-level authentication process to prevent unauthorized access. A legal team must collaborate with the development team, researchers, HCPs, and cultural/linguistic experts to ensure all legal matters are addressed and communicated clearly to users. Healthcare organizations must also be transparent about data accountability, encompassing both the organizations and the users involved.

ALiCon introduces a new set of healthcare competencies for paradigmatic navigators who can manage AI-mediated dialogues. The curricula must include AI literacy, focusing on how HCPs can oversee AI development and integrate it into patient-centered care. The empirical indicators suggested clinical accuracy, improved patient outcomes, and an enhanced healthcare delivery system. HCPs will need to develop a new skill set focused on AI-mediated competency. This involves recognizing clinical translation accuracy and cultural significance. Patient education shifts from giving instructions (one-way information delivery) to intuitive dialogues (two-way system). HCPs can use AI-driven translation to transform complex patient education into linguistically and culturally appropriate conversations, thereby improving patient engagement and health literacy. In areas with limited or no multilingual staff, ALiCon-informed practice provides a steadfast alternative to ad hoc interpreters, ensuring meaningful clinical and legal standards are met, accurately assessing symptoms, and reducing the risk of misdiagnosis.

Future research can use ALiCon’s empirical concepts to measure the impact of the AI-MC center on health system efficiency and patient outcomes. For example, an intervention study could compare hospital length of stay and readmission rates for LEP patients served by ALiCon-integrated environments versus conventional translation methods (medical or ad hoc interpreters). Another system metric is a cost-benefit analysis of AI-MC compared with human-only medical interpreters. 

There is a vital need to evaluate the psychometric properties of AI translation platforms. Other scales to establish reliability include patient and HCP trust in AI-MC and acceptance of AI-driven linguistic tools. Future studies should employ deductive approaches, such as a mixed-methods design, to explore human-AI interaction and qualitatively understand how HCPs maintain empathy while using a digital mediator. Intuitive dialogue accuracy can be quantitatively compared to medical interpreters in future investigations. 

ALiCon offers practical insights on when to use AI communication tools and how to maintain patient trust during AI-mediated interactions. This promotes mutual understanding between HCPs and patients and supports collaboration to achieve the desired patient outcomes. Healthcare administrators and developers can use ALiCon theory to outline critical boundaries and necessary safeguards when designing and implementing AI translation and communication platforms in clinical workflows. ALiCon is a newly constructed MRT; its proposed relationships regarding clinical safety and healthcare equity must be treated as testable hypotheses for prospective empirical validation. 

Limitations

While ALiCon offers a promising framework, its implementation faces barriers, including cost, workforce constraints, and users’ trust. In some cases, AI-mediated translation may not be applicable. It may not recognize and appropriately translate sensitive clinical dialogues that require empathy and cultural nuance, such as end-of-life discussions, delivering bad news, or psychiatric evaluations, where emotional and relational effects outweigh literal linguistic precision. In addition, in highly specialized medical subspecialties, AI translation errors or contextual hallucinations pose significant clinical safety risks, necessitating human medical interpreter oversight rather than full AI mediation. 

Other limitations of the theory development article using Walker and Avant’s framework include its reliance on existing literature. Although this approach integrates various conceptual philosophies, the resulting propositions remain theoretical constructs that require empirical validation through future clinical trials. While the author used systematic search parameters across different databases, selection bias may remain because of search-language constraints and date ranges. Consequently, the author may have excluded relevant findings from non-indexed journals or emerging technical reports. Moreover, the propositions of the ALiCon framework may vary significantly across different healthcare environments, clinical specialties, and patient demographic groups. Factors such as digital health literacy, LC, and regional AI regulatory environments may limit direct generalizability to resource-constrained settings or highly specialized acute care contexts.

AI's rapid evolution means the theory must remain flexible as the technology advances. The technology must continually adapt to the users' needs (i.e., HCPs and patients), requiring ongoing technical support and evaluation to improve functionality and efficiency. For ALiCon to truly advance health equity, the AI-driven technology must be accessible across all areas of healthcare, not only in select facilities with the capacity to implement and maintain the system.

## Conclusions

The ALiCon theory highlights an essential convergence of demographic shifts and technological advances. By positioning the HCP-patient relationship within AI-human integrative adaptation, ALiCon offers a practical approach to the challenges of linguistic discordance. It encourages HCPs to leverage AI-driven technology to support patient safety and uphold human dignity, helping close health disparities for millions of individuals navigating the healthcare system in languages other than English.

The theory presented is still in its early stages of development. It aims to fill a critical gap in understanding how to apply AI in a culturally and linguistically relevant manner for patient communication and engagement. The theory emphasizes involving multilingual HCPs in developing and refining AI tools for healthcare applications, recognizing their expertise as crucial to AI success. The ALiCon framework provides a theoretical basis for investigating how AI-MC may influence patient safety and health equity in future empirical studies.
